# Crystal structure of (±)-(1*SR*,5*SR*,6*SR*,7*SR*,10*SR*,11*SR*,13*SR*)-13-benz­yloxy-7-meth­oxy­meth­oxy-11,15,18,18-tetra­methyl-3-oxo-2,4-dioxa­tetra­cyclo­[12.3.1.0^1,5^.0^6,11^]octa­deca-14,16-dien-10-yl benzoate

**DOI:** 10.1107/S2056989015007136

**Published:** 2015-04-18

**Authors:** Takeshi Oishi, Keisuke Fukaya, Yu Yamaguchi, Tomoya Sugai, Ami Watanabe, Takaaki Sato, Noritaka Chida

**Affiliations:** a School of Medicine, Keio University, Hiyoshi 4-1-1, Kohoku-ku, Yokohama 223-8521, Japan; b Department of Applied Chemistry, Faculty of Science and Technology, Keio University, Hiyoshi 3-14-1, Kohoku-ku, Yokohama 223-8522, Japan

**Keywords:** crystal structure, hydrogen bonds, taxane skeleton, paclitaxel, hydrogen bonding, C—H⋯π inter­actions

## Abstract

In the title compound, C_36_H_42_O_8_, the dioxolane ring adopts a twist conformation; the two adjacent C atoms deviate alternately from the mean plane of other atoms by −0.287 (5) and 0.174 (5) Å. The cyclo­hexane, cyclo­hexa­diene and central cyclo­octane rings show chair, half-chair and boat–chair forms, respectively. As a result of the strained ring system, the tetra­subsituted olefin in the cyclo­hexa­diene is skewed from an ideal planar structure. In the crystal, C—H⋯O hydrogen bonds connect the mol­ecules into a sheet parallel to (100). The sheets are further linked by other weak C—H⋯O and C—H⋯π inter­actions, forming a three-dimensional network.

## Abstract 2

In the title compound, the ring conformations of the tetra­cycle are twist, chair, half-chair and chair–boat forms. In the crystal, inter­molecular C—H⋯O and C—H⋯π inter­actions link mol­ecules to construct a three-dimensional architecture.

## Chemical context   

**Figure 1:**
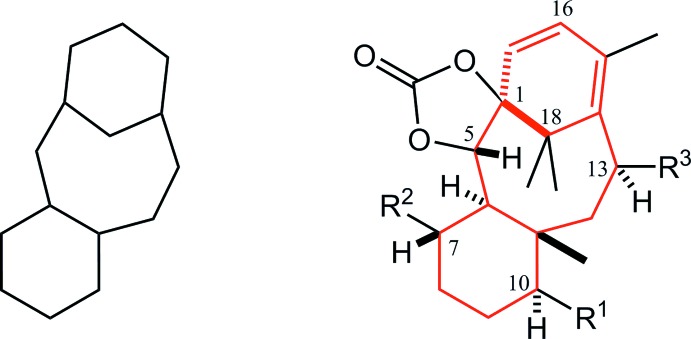
Left: Structure of the tri­cyclo­[9.3.1.0^3,8^]penta­decane (taxane) skeleton; Right: The title compound, indicating the taxane skeleton with red lines. *R* ^1^ = OC(=O)Ph, *R* ^2^ = OCH_2_OCH_3_, *R* ^3^ = OCH_2_Ph.

Paclitaxel is a well-known natural diterpenoid containing a taxane framework (tri­cyclo­[9.3.1.0^3,8^]penta­decane; Fig. 1 ▸), with a potent anti­tumor activity (Wall & Wani, 1995 ▸). The complicated structure and significant bioactivity have attracted chemical and medicinal inter­est. Previously, we have reported the crystal structures of the precursor for cyclization to build the taxane skeleton (Oishi, Yamaguchi *et al.*, 2015 ▸), and cyclized compounds (Oishi, Fukaya *et al.*, 2015 ▸) obtained in the synthetic study of paclitaxel. The title compound was afforded by further manipulation of functional groups of the cyclized compounds (Fukaya *et al.*, 2015 ▸).
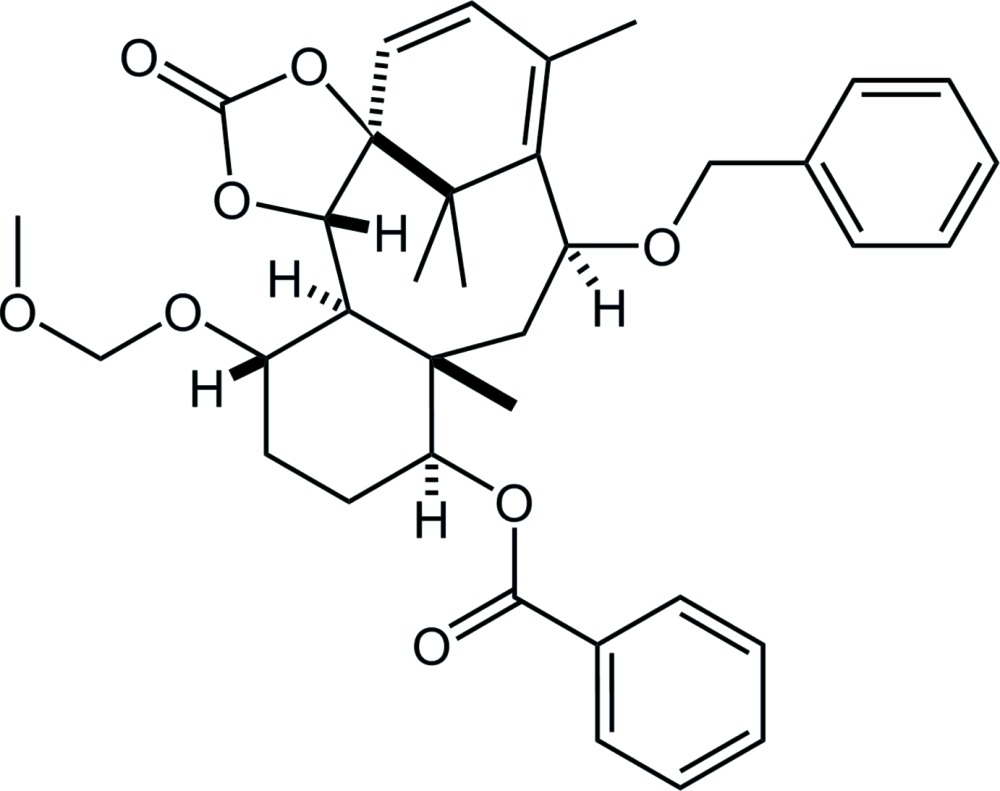


## Structural commentary   

**Figure 2:**
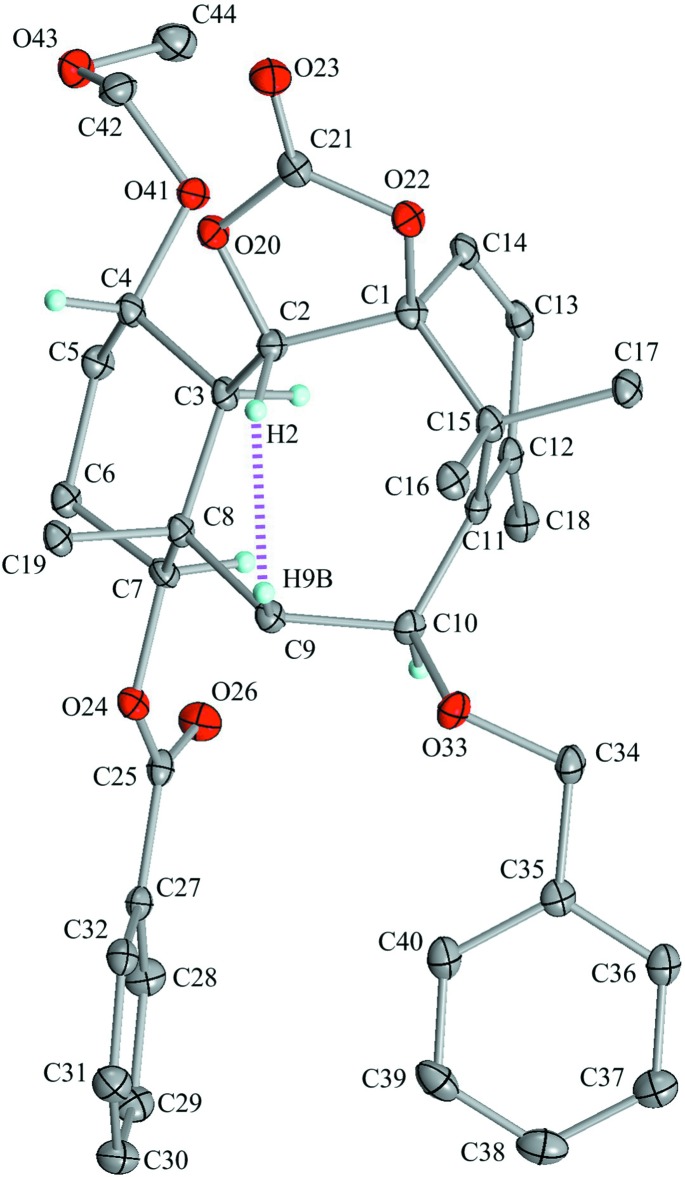
The mol­ecular structure of the title compound with the atom labeling. Displacement ellipsoids are drawn at the 30% probability level. The purple dotted line indicates the intra­molecular short contact. For clarity, only the H atoms attached to the chiral C atoms and related to the short contact are shown.

The mol­ecular structure of the title compound is shown in Fig. 2 ▸. The dioxolane ring (C1/C2/O20/C21/O22) adopts a twist form with puckering parameters of *Q*(2) = 0.272 (2) Å and *ϕ*(2) = 58.3 (5)°. Atoms C1 and C2 deviate from the mean plane of the other atoms by −0.287 (5) and 0.174 (5) Å, respectively. The cyclo­hexane ring (C3–C8) adopts a chair form with puckering parameters of *Q* = 0.590 (2) Å, *θ* = 10.97 (19)°, *ϕ* = 294.8 (12)°, *Q*(2) = 0.110 (2) Å and *Q*(3) = 0.579 (2) Å. The large substituents (C3—C2, C7—O24 and C8—C9) are in equatorial positions, while the meth­oxy­meth­oxy group (C4–O41) is slightly tilted from the ideal equatorial position with an angle to the *Cremer & Pople plane* of 59.01 (14)°.

The cyclo­hexa­diene ring (C1/C14/C13/C12/C11/C15) adopts a half-boat form with puckering parameters of *Q* = 0.598 (2) Å, *θ* = 115.68 (19)°, *ϕ* = 131.4 (3)°, *Q*(2) = 0.539 (2)° and *Q*(3) = 0.259 (2)°. The tetra­substituted olefin (C10/C15/C11=C12/C13/C18) is skewed from an ideal planar structure as a result of the strain in the fused-ring system, the C10—C11=C12—C18, C15—C11=C12—C13, C10—C11=C12—C13 and C15—C11=C12—C18 torsion angles being −19.5 (3), −18.4 (3), 150.34 (18) and 171.80 (18)°, respectively. The dihedral angle between the C10/C11/C15 and C18/C12/C13 planes is 26.4 (3)°. The other olefin (C12/C13=C14/C1) slightly deviates from planarity with a C12—C13=C14—C1 torsion angle of 9.1 (3)°. The diene moiety shows a C11=C12—C13=C14 torsion angle of −17.7 (3)°. The central cyclo­octane ring (C1–C3/C8–C11/C15) adopts a boat-chair form with puckering parameters of *Q* = 1.182 (2) Å, *Q*(2) = 0.897 (2) Å, *ϕ*(2) = 179.75 (15)°, *Q*(3) = 0.627 (2) Å, *ϕ*(3) = 2.7 (2)° and *Q*(4) = 0.441 (2) Å. There is an intra­molecular short contact of 1.98 Å between atoms H2 and H9*B* (Fig. 2 ▸).

## Supra­molecular features   

**Figure 3:**
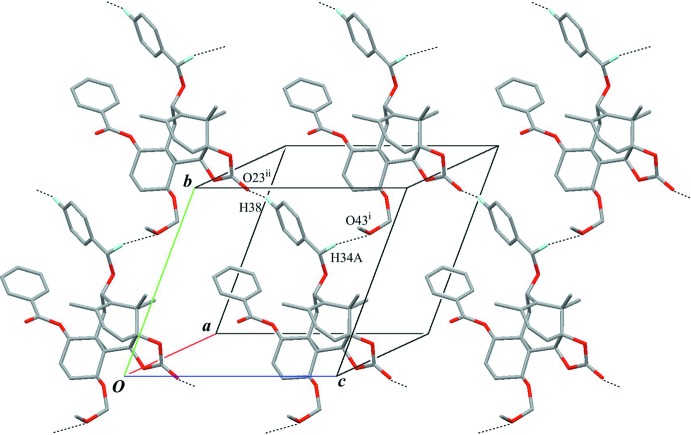
A partial packing view showing a sheet parallel to (100). Black dashed lines indicate the inter­molecular C—H⋯O inter­actions. Only H atoms involved in hydrogen bonds are shown for clarity. [Symmetry codes: (i) *x*, *y* + 1, *z*; (ii) *x*, *y* + 1, *z* − 1.]

**Figure 4:**
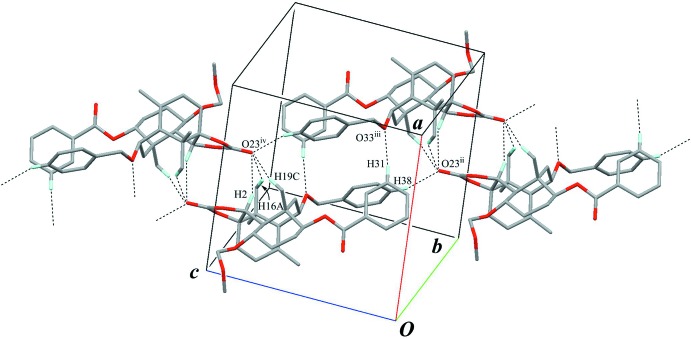
A packing diagram showing the connections between enanti­omers. Black dashed lines indicate the inter­molecular C—H⋯O inter­actions. Only H atoms involved in hydrogen bonds are shown for clarity. [Symmetry codes: (ii) *x*, *y* + 1, *z* − 1; (iii) −*x* + 1, −*y* + 1, −*z* + 1; (iv) −*x* + 1, −*y*, −*z* + 2.]

Inter­molecular C—H⋯O inter­actions (C34—H34*A*⋯O43^i^ and C38—H38⋯O23^ii^; Table 1 ▸ and Fig. 3 ▸) lead to the formation of a sheet parallel to (100). These sheets are further linked through weak inter­molecular C—H⋯O and C—H⋯π inter­actions (C31—H31⋯O33^iii^, C2—H2⋯O23^iv^, C16—H16*A*⋯O23^iv^, C19—H19*C*⋯O23^iv^ and C18—H18*C*⋯*Cg* ^v^; Table 1 ▸, Figs. 4 ▸ and 5 ▸) into a three-dimensional network.

## Database survey   

**Figure 6:**
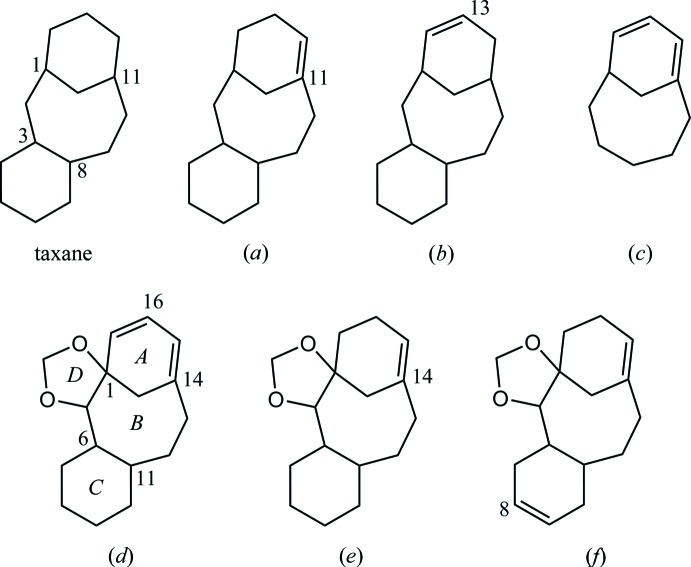
Core structures for database survey; tri­cyclo­[9.3.1.0^3,8^]penta­decane (taxane) and its (*a*) 11-ene and (*b*) 13-ene derivatives, (*c*) bi­cyclo­[5.3.1]undeca-7,9-diene, (*d*) the tetra­cyclic core of the title compound with ring labelling and (*e*) its di­hydro derivative and (*f*) the regioisomer of olefin. The ring-fusion geometries are similar to the title compound in each of the related structures, as *cis*-*AB*, *trans*-*BC* and *trans*-*BD*.

In the Cambridge Structural Database (CSD, Version 5.36, November 2014; Groom & Allen, 2014 ▸), 85 structures containing a tri­cyclo­[9.3.1.0^3,8^]penta­dec-11-ene skeleton, (*a*), are registered (Fig. 6 ▸). These include a large number of paclitaxels and its analogues, and one compound (NEGBOQ; Poujol *et al.*, 1997 ▸) containing a 2,4-dioxa­tetra­cyclo­[12.3.1.0^1,5^.0^6,11^]octa­dec-14-ene skeleton, (*e*), which is a di­hydro derivative for the tetra­cyclic core of the title compound, (*d*). Another related structure (SOJWOD; Paquette & Zhao, 1998 ▸) containing a tri­cyclo­[9.3.1.0^3,8^]penta­dec-13-ene skeleton, (*b*), has also been reported.

On the other hand, there are two related structures (GOQBET and GOQBIX; Keil *et al.*, 1994 ▸) containing a bi­cyclo­[5.3.1]undeca-7,9-diene skeleton, (*c*). Additionally, related tetra­cyclic taxoid (ILIQUP; Ohba *et al.*, 2003 ▸) and cyclic precursors for a taxane framework (NOTROF; Oishi, Yamaguchi *et al.*, 2015 ▸) were obtained in our previous study. Furthermore, the structures of the three related tetra­cyclic compounds have been reported (Oishi, Fukaya *et al.*, 2015 ▸). There are other crystalline compounds, closely related to the title compound with 2,4-dioxa­tetra­cyclo­[12.3.1.0^1,5^.0^6,11^]octa­deca-8,14-diene skeleton, (*f*) (Nicolaou, Ueno *et al.*, 1995 ▸; Nicolaou, Yang *et al.*, 1995 ▸), but they have not been deposited in the CSD.

## Synthesis and crystallization   

The title compound was provided in a synthetic study on paclitaxel (Fukaya *et al.*, 2015 ▸). The cyclo­hexa­diene unit (C1/C14/C13/C12/C11/C15) was synthesized according to the reported procedure (Nicolaou, Liu *et al.*, 1995 ▸), and coupled with the substituted cyclo­hexane unit (C3–C8) prepared from 3-methyl­anisole by a Shapiro reaction (Nicolaou, Liu *et al.*, 1995 ▸). A cyclization reaction followed by further manipulations of the functional groups afforded the title compound. Purification was carried out by silica gel chromatography, and colorless crystals were obtained from a benzene solution under a pentane-saturated atmosphere by slow evaporation at ambient temperature.

## Refinement   

Crystal data, data collection and structure refinement details are summarized in Table 2 ▸. C-bound H atoms were positioned geometrically with C—H = 0.95–1.00 Å, and constrained to ride on their parent atoms with *U*_iso_(H) = 1.2*U*_eq_(C) or 1.5*U*_eq_(methyl C).

## Supplementary Material

Crystal structure: contains datablock(s) global, I. DOI: 10.1107/S2056989015007136/is5396sup1.cif

Structure factors: contains datablock(s) I. DOI: 10.1107/S2056989015007136/is5396Isup2.hkl

CCDC reference: 1058739

Additional supporting information:  crystallographic information; 3D view; checkCIF report

## Figures and Tables

**Figure 5:**
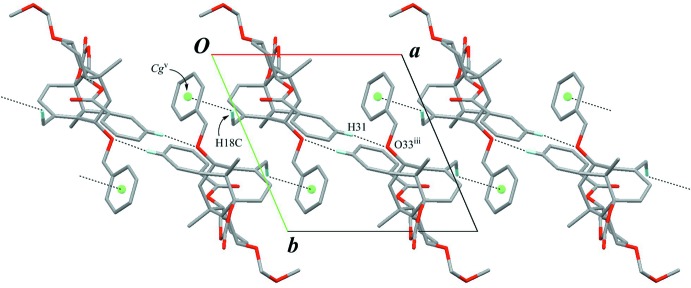
A packing diagram viewed down the *c* axis. Black dashed lines indicate the inter­molecular C—H⋯O and C—H⋯π inter­actions. *Cg* is the centroid of the C35–C40 benzene ring. Only H atoms involved in hydrogen bonds are shown for clarity. [Symmetry codes: (iii) −*x* + 1, −*y* + 1, −*z* + 1; (v) −*x*, −*y* + 1, −*z* + 1.]

**Table 1:** Hydrogen-bond geometry (, ) *Cg* is the centroid of the C35C40 benzene ring.

*D*H*A*	*D*H	H*A*	*D* *A*	*D*H*A*
C34H34*A*O43^i^	0.99	2.42	3.377(3)	163
C38H38O23^ii^	0.95	2.44	3.295(3)	149
C31H31O33^iii^	0.95	2.49	3.426(3)	168
C2H2O23^iv^	1.00	2.51	3.433(3)	153
C16H16*A*O23^iv^	0.98	2.53	3.357(3)	142
C19H19*C*O23^iv^	0.98	2.54	3.477(3)	160
C18H18*C* *Cg* ^v^	0.98	2.89	3.492(3)	121

Symmetry codes: (i) 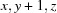; (ii) 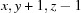; (iii) 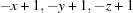; (iv) 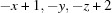; (v) 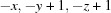.

**Table 2:** Experimental details

Crystal data	
Chemical formula	C_36_H_42_O_8_
*M*_r_	602.69
Crystal system, space group	Triclinic, *P* 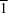
Temperature (K)	90
*a*, *b*, *c* ()	10.9358(6), 11.6121(6), 13.6833(7)
, , ()	72.148(2), 86.447(2), 66.766(2)
*V* (^3^)	1516.36(14)
*Z*	2
Radiation type	Mo *K*
(mm^1^)	0.09
Crystal size (mm)	0.32 0.27 0.16

Data collection	
Diffractometer	Bruker D8 Venture
Absorption correction	Multi-scan (*SADABS*; Bruker, 2014 ▸)
*T*_min_, *T*_max_	0.97, 0.98
No. of measured, independent and observed [*I* > 2(*I*)] reflections	27885, 5346, 4078
*R*_int_	0.052
(sin /)_max_ (^1^)	0.595

Refinement	
*R*[*F* ^2^ > 2(*F* ^2^)], *wR*(*F* ^2^), *S*	0.047, 0.120, 1.04
No. of reflections	5346
No. of parameters	402
H-atom treatment	H-atom parameters constrained
_max_, _min_ (e ^3^)	0.59, 0.23

Computer programs: *APEX2* and *SAINT* (Bruker, 2014 ▸), *SHELXS2013* (Sheldrick, 2008 ▸), *SHELXL2014* (Sheldrick, 2015 ▸), *Mercury* (Macrae *et al.*, 2006 ▸), *publCIF* (Westrip, 2010 ▸) and *PLATON* (Spek, 2009 ▸).

## supplementary crystallographic information

### Crystal data

**Table:**

C_36_H_42_O_8_	*F*(000) = 644
*M**_r_* = 602.69	*D*_x_ = 1.320 Mg m^−^^3^
Triclinic, *P*1	Melting point: 465.2 K
*a* = 10.9358 (6) Å	Mo *K*α radiation, λ = 0.71073 Å
*b* = 11.6121 (6) Å	Cell parameters from 9212 reflections
*c* = 13.6833 (7) Å	θ = 2.2–25.0°
α = 72.148 (2)°	µ = 0.09 mm^−^^1^
β = 86.447 (2)°	*T* = 90 K
γ = 66.766 (2)°	Prism, colorless
*V* = 1516.36 (14) Å^3^	0.32 × 0.27 × 0.16 mm
*Z* = 2	

### Data collection

**Table:**

Bruker D8 Venture diffractometer	5346 independent reflections
Radiation source: fine-focus sealed tube	4078 reflections with *I* > 2σ(*I*)
Multilayered confocal mirror monochromator	*R*_int_ = 0.052
Detector resolution: 8.333 pixels mm^-1^	θ_max_ = 25.0°, θ_min_ = 2.1°
φ and ω scans	*h* = −13→13
Absorption correction: multi-scan (*SADABS*; Bruker, 2014)	*k* = −13→13
*T*_min_ = 0.97, *T*_max_ = 0.98	*l* = −16→16
27885 measured reflections	

### Refinement

**Table:**

Refinement on *F*^2^	Primary atom site location: structure-invariant direct methods
Least-squares matrix: full	Secondary atom site location: difference Fourier map
*R*[*F*^2^ > 2σ(*F*^2^)] = 0.047	Hydrogen site location: inferred from neighbouring sites
*wR*(*F*^2^) = 0.120	H-atom parameters constrained
*S* = 1.04	*w* = 1/[σ^2^(*F*_o_^2^) + (0.0504*P*)^2^ + 1.0416*P*] where *P* = (*F*_o_^2^ + 2*F*_c_^2^)/3
5346 reflections	(Δ/σ)_max_ = 0.001
402 parameters	Δρ_max_ = 0.59 e Å^−^^3^
0 restraints	Δρ_min_ = −0.23 e Å^−^^3^

### Special details

**Table:**

Experimental. *M*.p. 462.2–465.2 K (not corrected); IR (film): 2940, 1806, 1716, 1274, 1109, 1043, 713 cm^-1^; ^1^H NMR (500 MHz, CDCl_3_): *δ* (p.p.m.) 8.03 (dd, *J* = 8.3, 1.2 Hz, 2H), 7.61 (tt, *J* = 7.5, 1.2 Hz, 1H), 7.49 (ddd, *J* = 8.3, 7.5, 1.7 Hz, 2H), 7.23–7.12 (m, 5H), 6.17 (d, *J* = 9.2 Hz, 1H), 5.63 (d, *J* = 9.2 Hz, 1H), 4.94 (d, *J* = 4.9 Hz, 1H), 4.90 (dd, *J* = 11.3, 5.2 Hz, 1H), 4.75 (d, *J* = 6.9 Hz, 1H), 4.65 (dd, *J* = 11.7, 5.4 Hz, 1H), 4.50 (d, *J* = 6.9 Hz, 1H), 4.47 (d, *J* = 12.0 Hz, 1H), 4.24 (d, *J* = 12.0 Hz, 1H), 3.70 (ddd, *J* = 10.5, 10.5, 4.9 Hz, 1H), 3.33 (s, 3H), 2.26 (dddd, *J* = 13.4, 5.0, 4.9, 2.6 Hz, 1H), 2.12 (dd, *J* = 15.9, 5.4 Hz, 1H), 2.04 (dd, *J* = 10.5, 4.9 Hz, 1H), 1.99 (dd, *J* = 15.9, 11.7 Hz, 1H), 1.91–1.85 (m, 1H), 1.84–1.73 (m, 1H), 1.80 (s, 3H), 1.58 (s, 3H), 1.50 (s, 3H), 1.35–1.24 (m, 1H), 1.18 (s, 3H); ^13^C NMR (125 MHz, CDCl_3_): *δ* (p.p.m.) 165.9 (C), 154.6 (C), 138.3 (C), 138.2 (C), 137.8 (C), 135.5 (CH), 133.5 (CH), 130.6 (CH), 130.1 (C), 129.8 (CH), 128.7 (CH), 128.5 (CH), 127.7 (CH), 127.4 (CH), 97.6 (CH_2_), 93.1 (C), 79.7 (CH), 74.2 (CH), 74.1 (CH), 73.4 (CH), 69.9 (CH_2_), 56.0 (CH_3_), 45.7 (CH), 42.8 (C), 39.8 (CH_2_), 37.9 (C), 32.5 (CH_2_), 29.5 (CH_3_), 25.8 (CH_2_), 19.33 (CH_3_), 19.27 (CH_3_), 17.7 (CH_3_); HRMS (ESI): calcd for C_36_H_42_O_8_Na^+^ [*M*+Na]^+^ 625.2777, found 625.2777.
Geometry. All e.s.d.'s (except the e.s.d. in the dihedral angle between two l.s. planes) are estimated using the full covariance matrix. The cell e.s.d.'s are taken into account individually in the estimation of e.s.d.'s in distances, angles and torsion angles; correlations between e.s.d.'s in cell parameters are only used when they are defined by crystal symmetry. An approximate (isotropic) treatment of cell e.s.d.'s is used for estimating e.s.d.'s involving l.s. planes.
Refinement. Refinement of *F*^2^ against ALL reflections. The weighted *R*-factor *wR* and goodness of fit *S* are based on *F*^2^, conventional *R*-factors *R* are based on *F*, with *F* set to zero for negative *F*^2^. The threshold expression of *F*^2^ > σ(*F*^2^) is used only for calculating *R*-factors(gt) *etc*. and is not relevant to the choice of reflections for refinement. *R*-factors based on *F*^2^ are statistically about twice as large as those based on *F*, and *R*- factors based on ALL data will be even larger.Problematic one reflection with |*I*(obs)-*I*(calc)|/σW(*I*) greater than 10 (–2 3 1) has been omitted in the final refinement.

### Fractional atomic coordinates and isotropic or equivalent isotropic displacement parameters (Å^2^)

**Table:**

	*x*	*y*	*z*	*U*_iso_*/*U*_eq_	
C1	0.2090 (2)	0.1750 (2)	0.91158 (15)	0.0210 (5)	
C2	0.3204 (2)	0.0817 (2)	0.86310 (15)	0.0198 (5)	
H2	0.3938	0.1143	0.8498	0.024*	
C3	0.2821 (2)	0.0698 (2)	0.76154 (15)	0.0184 (4)	
H3	0.1858	0.1295	0.7447	0.022*	
C4	0.2914 (2)	−0.0675 (2)	0.76837 (15)	0.0207 (5)	
H4	0.3848	−0.1344	0.7883	0.025*	
C5	0.2414 (2)	−0.0651 (2)	0.66551 (15)	0.0225 (5)	
H5B	0.2627	−0.1569	0.6672	0.027*	
H5A	0.1431	−0.0182	0.6578	0.027*	
C6	0.2999 (2)	0.0002 (2)	0.57150 (15)	0.0214 (5)	
H6A	0.3951	−0.0565	0.5704	0.026*	
H6B	0.2523	0.0104	0.5082	0.026*	
C7	0.2877 (2)	0.1337 (2)	0.57361 (15)	0.0191 (5)	
H7	0.191	0.191	0.5717	0.023*	
C8	0.3583 (2)	0.1246 (2)	0.67110 (15)	0.0183 (4)	
C9	0.3595 (2)	0.2595 (2)	0.66630 (16)	0.0199 (5)	
H9B	0.4203	0.242	0.7246	0.024*	
H9A	0.4028	0.2871	0.6028	0.024*	
C10	0.2330 (2)	0.3811 (2)	0.66790 (16)	0.0198 (5)	
H10	0.1778	0.413	0.602	0.024*	
C11	0.1476 (2)	0.35654 (19)	0.75632 (16)	0.0197 (5)	
C12	0.0331 (2)	0.3454 (2)	0.73994 (15)	0.0204 (5)	
C13	−0.0137 (2)	0.2618 (2)	0.82427 (16)	0.0230 (5)	
H13	−0.1038	0.2705	0.8228	0.028*	
C14	0.0722 (2)	0.1738 (2)	0.90249 (16)	0.0229 (5)	
H14	0.0479	0.1105	0.9525	0.027*	
C15	0.1993 (2)	0.3171 (2)	0.86995 (15)	0.0207 (5)	
C16	0.3299 (2)	0.3293 (2)	0.88962 (16)	0.0229 (5)	
H16B	0.3555	0.2938	0.9636	0.034*	
H16C	0.317	0.4222	0.865	0.034*	
H16A	0.4005	0.2795	0.8529	0.034*	
C17	0.0958 (2)	0.4076 (2)	0.92429 (16)	0.0243 (5)	
H17A	0.0079	0.4077	0.9138	0.036*	
H17B	0.092	0.4974	0.8955	0.036*	
H17C	0.1216	0.3753	0.9981	0.036*	
C18	−0.0437 (2)	0.3934 (2)	0.63817 (16)	0.0255 (5)	
H18A	−0.0215	0.4638	0.5908	0.038*	
H18B	−0.1396	0.4273	0.648	0.038*	
H18C	−0.0202	0.3204	0.6094	0.038*	
C19	0.5062 (2)	0.0313 (2)	0.68131 (16)	0.0211 (5)	
H19B	0.5135	−0.0561	0.6827	0.032*	
H19C	0.5482	0.0244	0.7452	0.032*	
H19A	0.5512	0.0659	0.6225	0.032*	
O20	0.36857 (14)	−0.04110 (14)	0.94740 (10)	0.0226 (3)	
C21	0.3358 (2)	−0.0133 (2)	1.03550 (16)	0.0229 (5)	
O22	0.25645 (14)	0.11453 (14)	1.01974 (10)	0.0241 (3)	
O23	0.37257 (15)	−0.09211 (15)	1.11907 (11)	0.0301 (4)	
O24	0.34533 (13)	0.19559 (14)	0.48470 (10)	0.0200 (3)	
C25	0.2682 (2)	0.2569 (2)	0.39610 (15)	0.0204 (5)	
O26	0.16003 (15)	0.25445 (15)	0.38570 (11)	0.0290 (4)	
C27	0.3292 (2)	0.3303 (2)	0.31322 (15)	0.0192 (4)	
C28	0.2699 (2)	0.3799 (2)	0.21389 (16)	0.0269 (5)	
H28	0.1964	0.362	0.1999	0.032*	
C29	0.3180 (2)	0.4553 (2)	0.13538 (17)	0.0300 (5)	
H29	0.2785	0.4879	0.0673	0.036*	
C30	0.4233 (2)	0.4832 (2)	0.15603 (17)	0.0299 (5)	
H30	0.4553	0.5363	0.1023	0.036*	
C31	0.4824 (2)	0.4342 (2)	0.25464 (17)	0.0279 (5)	
H31	0.5552	0.4534	0.2684	0.033*	
C32	0.4363 (2)	0.3575 (2)	0.33317 (16)	0.0218 (5)	
H32	0.4776	0.3234	0.4008	0.026*	
O33	0.28434 (14)	0.47725 (14)	0.66845 (11)	0.0239 (3)	
C34	0.1863 (2)	0.6092 (2)	0.63828 (16)	0.0246 (5)	
H34A	0.1914	0.653	0.6887	0.029*	
H34B	0.0964	0.6075	0.6389	0.029*	
C35	0.2054 (2)	0.6865 (2)	0.53358 (16)	0.0217 (5)	
C36	0.1191 (2)	0.8177 (2)	0.49309 (17)	0.0260 (5)	
H36	0.0481	0.8562	0.5317	0.031*	
C37	0.1356 (2)	0.8926 (2)	0.39732 (17)	0.0313 (5)	
H37	0.0773	0.9827	0.3712	0.038*	
C38	0.2363 (2)	0.8373 (3)	0.33917 (18)	0.0346 (6)	
H38	0.2465	0.8885	0.2727	0.041*	
C39	0.3217 (2)	0.7074 (3)	0.37825 (18)	0.0334 (6)	
H39	0.3909	0.6689	0.3383	0.04*	
C40	0.3080 (2)	0.6325 (2)	0.47451 (17)	0.0269 (5)	
H40	0.3688	0.5433	0.501	0.032*	
O41	0.20301 (14)	−0.09407 (14)	0.84612 (10)	0.0234 (3)	
C42	0.2292 (2)	−0.2270 (2)	0.89614 (17)	0.0287 (5)	
H42B	0.194	−0.2359	0.9653	0.034*	
H42A	0.3269	−0.2778	0.9054	0.034*	
O43	0.17233 (17)	−0.28080 (15)	0.84205 (12)	0.0328 (4)	
C44	0.0307 (2)	−0.2202 (2)	0.83398 (19)	0.0352 (6)	
H44C	−0.0029	−0.2401	0.9026	0.053*	
H44A	−0.0046	−0.254	0.7899	0.053*	
H44B	0.0019	−0.1246	0.8039	0.053*	

### Atomic displacement parameters (Å^2^)

**Table:**

	*U*^11^	*U*^22^	*U*^33^	*U*^12^	*U*^13^	*U*^23^
C1	0.0266 (11)	0.0233 (12)	0.0175 (11)	−0.0112 (9)	0.0021 (9)	−0.0108 (9)
C2	0.0242 (11)	0.0197 (11)	0.0184 (11)	−0.0102 (9)	0.0022 (9)	−0.0081 (9)
C3	0.0212 (10)	0.0200 (11)	0.0184 (10)	−0.0088 (9)	0.0023 (8)	−0.0110 (9)
C4	0.0234 (11)	0.0212 (12)	0.0221 (11)	−0.0102 (9)	0.0056 (9)	−0.0118 (9)
C5	0.0293 (12)	0.0228 (12)	0.0238 (11)	−0.0148 (10)	0.0043 (9)	−0.0129 (9)
C6	0.0250 (11)	0.0233 (12)	0.0208 (11)	−0.0103 (9)	0.0021 (9)	−0.0124 (9)
C7	0.0210 (11)	0.0224 (11)	0.0182 (11)	−0.0104 (9)	0.0059 (9)	−0.0102 (9)
C8	0.0208 (11)	0.0191 (11)	0.0191 (10)	−0.0086 (9)	0.0026 (8)	−0.0108 (9)
C9	0.0224 (11)	0.0222 (12)	0.0201 (11)	−0.0115 (9)	0.0036 (9)	−0.0102 (9)
C10	0.0230 (11)	0.0192 (11)	0.0232 (11)	−0.0112 (9)	0.0006 (9)	−0.0105 (9)
C11	0.0204 (11)	0.0151 (11)	0.0264 (11)	−0.0057 (9)	0.0014 (9)	−0.0118 (9)
C12	0.0216 (11)	0.0181 (11)	0.0244 (11)	−0.0058 (9)	0.0037 (9)	−0.0137 (9)
C13	0.0203 (11)	0.0268 (12)	0.0281 (12)	−0.0094 (10)	0.0054 (9)	−0.0176 (10)
C14	0.0256 (12)	0.0237 (12)	0.0271 (12)	−0.0122 (10)	0.0105 (10)	−0.0168 (10)
C15	0.0224 (11)	0.0222 (12)	0.0228 (11)	−0.0097 (9)	0.0032 (9)	−0.0132 (9)
C16	0.0272 (12)	0.0264 (12)	0.0225 (11)	−0.0127 (10)	0.0007 (9)	−0.0143 (9)
C17	0.0287 (12)	0.0237 (12)	0.0256 (12)	−0.0107 (10)	0.0043 (9)	−0.0146 (10)
C18	0.0238 (11)	0.0270 (13)	0.0300 (12)	−0.0107 (10)	0.0004 (10)	−0.0136 (10)
C19	0.0236 (11)	0.0244 (12)	0.0206 (11)	−0.0104 (9)	0.0040 (9)	−0.0135 (9)
O20	0.0280 (8)	0.0228 (8)	0.0176 (7)	−0.0086 (7)	0.0013 (6)	−0.0086 (6)
C21	0.0209 (11)	0.0281 (13)	0.0234 (12)	−0.0109 (10)	0.0025 (9)	−0.0115 (10)
O22	0.0316 (8)	0.0251 (9)	0.0187 (8)	−0.0111 (7)	0.0022 (6)	−0.0112 (6)
O23	0.0337 (9)	0.0333 (9)	0.0214 (9)	−0.0122 (7)	−0.0010 (7)	−0.0064 (7)
O24	0.0216 (7)	0.0235 (8)	0.0181 (7)	−0.0104 (6)	0.0018 (6)	−0.0088 (6)
C25	0.0211 (11)	0.0186 (11)	0.0229 (11)	−0.0053 (9)	−0.0003 (9)	−0.0112 (9)
O26	0.0252 (9)	0.0362 (10)	0.0278 (8)	−0.0154 (7)	−0.0016 (7)	−0.0080 (7)
C27	0.0208 (11)	0.0173 (11)	0.0211 (11)	−0.0066 (9)	0.0018 (9)	−0.0095 (9)
C28	0.0292 (12)	0.0304 (13)	0.0271 (12)	−0.0163 (11)	−0.0025 (10)	−0.0098 (10)
C29	0.0380 (14)	0.0318 (14)	0.0219 (12)	−0.0148 (11)	−0.0017 (10)	−0.0084 (10)
C30	0.0365 (13)	0.0330 (13)	0.0246 (12)	−0.0198 (11)	0.0044 (10)	−0.0073 (10)
C31	0.0286 (12)	0.0316 (13)	0.0308 (13)	−0.0171 (11)	0.0036 (10)	−0.0130 (10)
C32	0.0218 (11)	0.0227 (12)	0.0209 (11)	−0.0065 (9)	−0.0013 (9)	−0.0092 (9)
O33	0.0247 (8)	0.0195 (8)	0.0317 (8)	−0.0110 (7)	−0.0002 (6)	−0.0101 (7)
C34	0.0248 (11)	0.0210 (12)	0.0322 (12)	−0.0088 (10)	0.0035 (10)	−0.0146 (10)
C35	0.0232 (11)	0.0249 (12)	0.0252 (11)	−0.0134 (9)	−0.0005 (9)	−0.0131 (9)
C36	0.0247 (12)	0.0270 (13)	0.0317 (13)	−0.0109 (10)	0.0004 (10)	−0.0153 (10)
C37	0.0319 (13)	0.0323 (14)	0.0318 (13)	−0.0165 (11)	−0.0032 (11)	−0.0065 (11)
C38	0.0395 (14)	0.0462 (16)	0.0291 (13)	−0.0300 (13)	0.0020 (11)	−0.0090 (12)
C39	0.0321 (13)	0.0495 (17)	0.0345 (14)	−0.0269 (13)	0.0136 (11)	−0.0221 (12)
C40	0.0232 (12)	0.0292 (13)	0.0371 (13)	−0.0128 (10)	0.0031 (10)	−0.0191 (11)
O41	0.0320 (8)	0.0214 (8)	0.0227 (8)	−0.0143 (7)	0.0076 (6)	−0.0108 (6)
C42	0.0376 (13)	0.0233 (13)	0.0267 (12)	−0.0149 (11)	0.0049 (10)	−0.0063 (10)
O43	0.0443 (10)	0.0288 (9)	0.0386 (9)	−0.0219 (8)	0.0153 (8)	−0.0207 (7)
C44	0.0416 (15)	0.0379 (15)	0.0379 (14)	−0.0252 (12)	0.0096 (11)	−0.0164 (12)

### Geometric parameters (Å, º)

**Table:**

C1—O22	1.457 (2)	C18—H18B	0.98
C1—C14	1.514 (3)	C18—H18C	0.98
C1—C15	1.533 (3)	C19—H19B	0.98
C1—C2	1.548 (3)	C19—H19C	0.98
C2—O20	1.454 (2)	C19—H19A	0.98
C2—C3	1.537 (3)	O20—C21	1.334 (2)
C2—H2	1.0	C21—O23	1.196 (3)
C3—C4	1.530 (3)	C21—O22	1.347 (3)
C3—C8	1.561 (3)	O24—C25	1.346 (2)
C3—H3	1.0	C25—O26	1.213 (2)
C4—O41	1.438 (2)	C25—C27	1.490 (3)
C4—C5	1.530 (3)	C27—C32	1.389 (3)
C4—H4	1.0	C27—C28	1.391 (3)
C5—C6	1.524 (3)	C28—C29	1.383 (3)
C5—H5B	0.99	C28—H28	0.95
C5—H5A	0.99	C29—C30	1.379 (3)
C6—C7	1.512 (3)	C29—H29	0.95
C6—H6A	0.99	C30—C31	1.382 (3)
C6—H6B	0.99	C30—H30	0.95
C7—O24	1.456 (2)	C31—C32	1.379 (3)
C7—C8	1.537 (3)	C31—H31	0.95
C7—H7	1.0	C32—H32	0.95
C8—C19	1.537 (3)	O33—C34	1.428 (2)
C8—C9	1.553 (3)	C34—C35	1.491 (3)
C9—C10	1.543 (3)	C34—H34A	0.99
C9—H9B	0.99	C34—H34B	0.99
C9—H9A	0.99	C35—C36	1.390 (3)
C10—O33	1.436 (2)	C35—C40	1.396 (3)
C10—C11	1.508 (3)	C36—C37	1.379 (3)
C10—H10	1.0	C36—H36	0.95
C11—C12	1.348 (3)	C37—C38	1.380 (3)
C11—C15	1.554 (3)	C37—H37	0.95
C12—C13	1.473 (3)	C38—C39	1.374 (4)
C12—C18	1.502 (3)	C38—H38	0.95
C13—C14	1.330 (3)	C39—C40	1.375 (3)
C13—H13	0.95	C39—H39	0.95
C14—H14	0.95	C40—H40	0.95
C15—C16	1.537 (3)	O41—C42	1.400 (3)
C15—C17	1.541 (3)	C42—O43	1.405 (3)
C16—H16B	0.98	C42—H42B	0.99
C16—H16C	0.98	C42—H42A	0.99
C16—H16A	0.98	O43—C44	1.421 (3)
C17—H17A	0.98	C44—H44C	0.98
C17—H17B	0.98	C44—H44A	0.98
C17—H17C	0.98	C44—H44B	0.98
C18—H18A	0.98		
			
O22—C1—C14	107.39 (16)	C15—C17—H17A	109.5
O22—C1—C15	112.26 (16)	C15—C17—H17B	109.5
C14—C1—C15	109.44 (17)	H17A—C17—H17B	109.5
O22—C1—C2	100.39 (15)	C15—C17—H17C	109.5
C14—C1—C2	115.12 (16)	H17A—C17—H17C	109.5
C15—C1—C2	111.91 (17)	H17B—C17—H17C	109.5
O20—C2—C3	114.77 (16)	C12—C18—H18A	109.5
O20—C2—C1	102.39 (15)	C12—C18—H18B	109.5
C3—C2—C1	116.91 (17)	H18A—C18—H18B	109.5
O20—C2—H2	107.4	C12—C18—H18C	109.5
C3—C2—H2	107.4	H18A—C18—H18C	109.5
C1—C2—H2	107.4	H18B—C18—H18C	109.5
C4—C3—C2	114.36 (16)	C8—C19—H19B	109.5
C4—C3—C8	112.76 (16)	C8—C19—H19C	109.5
C2—C3—C8	111.37 (16)	H19B—C19—H19C	109.5
C4—C3—H3	105.9	C8—C19—H19A	109.5
C2—C3—H3	105.9	H19B—C19—H19A	109.5
C8—C3—H3	105.9	H19C—C19—H19A	109.5
O41—C4—C3	104.79 (15)	C21—O20—C2	108.37 (16)
O41—C4—C5	109.40 (16)	O23—C21—O20	124.3 (2)
C3—C4—C5	109.75 (17)	O23—C21—O22	123.56 (19)
O41—C4—H4	110.9	O20—C21—O22	112.13 (18)
C3—C4—H4	110.9	C21—O22—C1	108.85 (15)
C5—C4—H4	110.9	C25—O24—C7	116.56 (15)
C6—C5—C4	114.71 (17)	O26—C25—O24	123.60 (19)
C6—C5—H5B	108.6	O26—C25—C27	123.88 (19)
C4—C5—H5B	108.6	O24—C25—C27	112.51 (17)
C6—C5—H5A	108.6	C32—C27—C28	119.65 (19)
C4—C5—H5A	108.6	C32—C27—C25	122.37 (18)
H5B—C5—H5A	107.6	C28—C27—C25	117.85 (19)
C7—C6—C5	110.62 (16)	C29—C28—C27	120.1 (2)
C7—C6—H6A	109.5	C29—C28—H28	120.0
C5—C6—H6A	109.5	C27—C28—H28	120.0
C7—C6—H6B	109.5	C30—C29—C28	120.0 (2)
C5—C6—H6B	109.5	C30—C29—H29	120.0
H6A—C6—H6B	108.1	C28—C29—H29	120.0
O24—C7—C6	110.82 (15)	C29—C30—C31	120.2 (2)
O24—C7—C8	108.01 (15)	C29—C30—H30	119.9
C6—C7—C8	112.06 (17)	C31—C30—H30	119.9
O24—C7—H7	108.6	C32—C31—C30	120.3 (2)
C6—C7—H7	108.6	C32—C31—H31	119.8
C8—C7—H7	108.6	C30—C31—H31	119.8
C19—C8—C7	110.94 (16)	C31—C32—C27	119.86 (19)
C19—C8—C9	104.78 (16)	C31—C32—H32	120.1
C7—C8—C9	111.49 (16)	C27—C32—H32	120.1
C19—C8—C3	110.89 (16)	C34—O33—C10	113.59 (15)
C7—C8—C3	104.62 (15)	O33—C34—C35	111.86 (17)
C9—C8—C3	114.26 (16)	O33—C34—H34A	109.2
C10—C9—C8	123.76 (17)	C35—C34—H34A	109.2
C10—C9—H9B	106.4	O33—C34—H34B	109.2
C8—C9—H9B	106.4	C35—C34—H34B	109.2
C10—C9—H9A	106.4	H34A—C34—H34B	107.9
C8—C9—H9A	106.4	C36—C35—C40	118.3 (2)
H9B—C9—H9A	106.5	C36—C35—C34	119.20 (19)
O33—C10—C11	113.16 (16)	C40—C35—C34	122.5 (2)
O33—C10—C9	103.42 (15)	C37—C36—C35	120.6 (2)
C11—C10—C9	114.58 (17)	C37—C36—H36	119.7
O33—C10—H10	108.5	C35—C36—H36	119.7
C11—C10—H10	108.5	C36—C37—C38	120.4 (2)
C9—C10—H10	108.5	C36—C37—H37	119.8
C12—C11—C10	119.94 (18)	C38—C37—H37	119.8
C12—C11—C15	117.22 (18)	C39—C38—C37	119.4 (2)
C10—C11—C15	121.82 (17)	C39—C38—H38	120.3
C11—C12—C13	118.68 (19)	C37—C38—H38	120.3
C11—C12—C18	126.2 (2)	C38—C39—C40	120.7 (2)
C13—C12—C18	114.38 (18)	C38—C39—H39	119.6
C14—C13—C12	118.85 (19)	C40—C39—H39	119.6
C14—C13—H13	120.6	C39—C40—C35	120.5 (2)
C12—C13—H13	120.6	C39—C40—H40	119.8
C13—C14—C1	119.8 (2)	C35—C40—H40	119.8
C13—C14—H14	120.1	C42—O41—C4	116.28 (16)
C1—C14—H14	120.1	O41—C42—O43	112.97 (18)
C1—C15—C16	112.74 (17)	O41—C42—H42B	109.0
C1—C15—C17	111.81 (17)	O43—C42—H42B	109.0
C16—C15—C17	104.00 (16)	O41—C42—H42A	109.0
C1—C15—C11	101.58 (15)	O43—C42—H42A	109.0
C16—C15—C11	117.71 (17)	H42B—C42—H42A	107.8
C17—C15—C11	109.19 (17)	C42—O43—C44	112.09 (17)
C15—C16—H16B	109.5	O43—C44—H44C	109.5
C15—C16—H16C	109.5	O43—C44—H44A	109.5
H16B—C16—H16C	109.5	H44C—C44—H44A	109.5
C15—C16—H16A	109.5	O43—C44—H44B	109.5
H16B—C16—H16A	109.5	H44C—C44—H44B	109.5
H16C—C16—H16A	109.5	H44A—C44—H44B	109.5
			
O22—C1—C2—O20	26.87 (18)	C3—C8—C9—C10	−51.5 (3)
C14—C1—C2—O20	−88.07 (19)	C8—C9—C10—O33	176.60 (17)
C15—C1—C2—O20	146.14 (16)	C8—C9—C10—C11	53.0 (3)
O22—C1—C2—C3	153.21 (16)	O33—C10—C11—C12	137.37 (19)
C14—C1—C2—C3	38.3 (3)	C9—C10—C11—C12	−104.4 (2)
C15—C1—C2—C3	−87.5 (2)	O33—C10—C11—C15	−54.5 (2)
O20—C2—C3—C4	4.3 (2)	C9—C10—C11—C15	63.8 (2)
C1—C2—C3—C4	−115.6 (2)	C10—C11—C12—C13	150.34 (18)
O20—C2—C3—C8	−124.98 (18)	C15—C11—C12—C13	−18.4 (3)
C1—C2—C3—C8	115.07 (19)	C10—C11—C12—C18	−19.5 (3)
C2—C3—C4—O41	58.5 (2)	C15—C11—C12—C18	171.80 (18)
C8—C3—C4—O41	−172.95 (15)	C11—C12—C13—C14	−17.7 (3)
C2—C3—C4—C5	175.84 (17)	C18—C12—C13—C14	153.33 (19)
C8—C3—C4—C5	−55.6 (2)	C12—C13—C14—C1	9.1 (3)
O41—C4—C5—C6	163.01 (17)	O22—C1—C14—C13	155.28 (18)
C3—C4—C5—C6	48.5 (2)	C15—C1—C14—C13	33.2 (2)
C4—C5—C6—C7	−50.2 (2)	C2—C1—C14—C13	−93.9 (2)
C5—C6—C7—O24	179.13 (16)	O22—C1—C15—C16	53.1 (2)
C5—C6—C7—C8	58.4 (2)	C14—C1—C15—C16	172.27 (16)
O24—C7—C8—C19	−65.5 (2)	C2—C1—C15—C16	−58.9 (2)
C6—C7—C8—C19	56.9 (2)	O22—C1—C15—C17	−63.6 (2)
O24—C7—C8—C9	50.9 (2)	C14—C1—C15—C17	55.5 (2)
C6—C7—C8—C9	173.24 (16)	C2—C1—C15—C17	−175.65 (16)
O24—C7—C8—C3	174.89 (15)	O22—C1—C15—C11	−179.97 (16)
C6—C7—C8—C3	−62.8 (2)	C14—C1—C15—C11	−60.82 (19)
C4—C3—C8—C19	−57.8 (2)	C2—C1—C15—C11	68.0 (2)
C2—C3—C8—C19	72.3 (2)	C12—C11—C15—C1	56.4 (2)
C4—C3—C8—C7	61.8 (2)	C10—C11—C15—C1	−112.1 (2)
C2—C3—C8—C7	−168.02 (16)	C12—C11—C15—C16	179.97 (18)
C4—C3—C8—C9	−175.95 (16)	C10—C11—C15—C16	11.5 (3)
C2—C3—C8—C9	−45.8 (2)	C12—C11—C15—C17	−61.8 (2)
C19—C8—C9—C10	−173.02 (18)	C10—C11—C15—C17	129.69 (19)
C7—C8—C9—C10	66.9 (2)	C3—C2—O20—C21	−149.52 (17)

### Hydrogen-bond geometry (Å, º)

Cg is the centroid of the C35–C40 benzene ring.

**Table:**

*D*—H···*A*	*D*—H	H···*A*	*D*···*A*	*D*—H···*A*
C34—H34*A*···O43^i^	0.99	2.42	3.377 (3)	163
C38—H38···O23^ii^	0.95	2.44	3.295 (3)	149
C31—H31···O33^iii^	0.95	2.49	3.426 (3)	168
C2—H2···O23^iv^	1.00	2.51	3.433 (3)	153
C16—H16*A*···O23^iv^	0.98	2.53	3.357 (3)	142
C19—H19*C*···O23^iv^	0.98	2.54	3.477 (3)	160
C18—H18*C*···*Cg*^v^	0.98	2.89	3.492 (3)	121

Symmetry codes: (i) *x*, *y*+1, *z*; (ii) *x*, *y*+1, *z*−1; (iii) −*x*+1, −*y*+1, −*z*+1; (iv) −*x*+1, −*y*, −*z*+2; (v) −*x*, −*y*+1, −*z*+1.
